# Ultrasound‐guided puncture into newborn rat brain

**DOI:** 10.1002/ibra.12103

**Published:** 2023-05-10

**Authors:** Rui‐Fang Ma, Ping‐Chieh Pao, Kun Zhang, Jin‐Xiang Liu, Lin Zhang

**Affiliations:** ^1^ Institute of Neuroscience Kunming Medical University Kunming Yunnan China; ^2^ Picower Institute for Learning and Memory, Department of Brain and Cognitive Sciences Massachusetts Institute of Technology Cambridge Massachusetts USA; ^3^ Institute of Ultrasound Shantou Ultrasonic Instrument Research Institute Co. Ltd. Shantou Guangdong China; ^4^ Department of Obstetrics, The International Peace Maternity and Child Health Hospital, School of Medicine Shanghai Jiao Tong University Shanghai China

**Keywords:** brain structure, cortex, hippocampus, lateral ventricle, striatum, ultrasonoscopy

## Abstract

Since the brain structure of neonatal rats was not fully formed during the first 4 days, it cannot be detected using ultrasound. The objective of this study was to investigate the use of ultrasound to guide puncture in the normal coronal brain structure and determine the puncture depth of the location of the cortex, hippocampus, lateral ventricle, and striatum of newborn rats of 5−15 days. The animal was placed in a prone position. The specific positions of the cortex, hippocampus, lateral ventricle, and striatum were measured under ultrasound. Then, the rats were punctured with a stereotaxic instrument, and dye was injected. Finally, the brains of rats were taken to make frozen sections to observe the puncture results. By ultrasound, the image of the cortex, hippocampus, lateral ventricle, and striatum of the rat can be obtained and the puncture depth of the cortex (8 days: 1.02 ± 0.12, 10 days: 1.02 ± 0.08, 13 days: 1.43 ± 0.05), hippocampus (8 days: 2.63 ± 0.07, 10 days: 2.77 ± 0.14, 13 days: 2.82 ± 0.09), lateral ventricle (8 days: 2.08 ± 0.04, 10 days: 2.26 ± 0.03, 13 days: 2.40 ± 0.06), and corpus striatum (8 days: 4.57 ± 0.09, 10 days: 4.94 ± 0.31, 13 days: 5.13 ± 0.10) can be accurately measured. The rat brain structure and puncture depth changed with the age of the rats. Ultrasound technology can not only clarify the brain structure characteristics of 5—15‐day‐old rats but also guide the puncture and injection of the rat brain structure. The results of this study laid the foundation for the future use of ultrasound in experimental animal models of neurological diseases.

## Graphical abstract

SD rats were injected with dye using a stereotaxic instrument, and after freezing, brain structures were cut to determine the status of the dye in the cortex, hippocampus, lateral ventricle, and striatum.

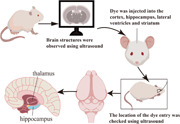

## 1 INTRODUCTION

Ultrasound has several well‐known advantages, including noninvasiveness, low cost, portability, safety, and fast real‐time imaging,^1^, ^2^ making it widely used in clinical practice. Most of the examinations are inseparable from ultrasound because it can examine patients from different angles and directions. In addition, ultrasound has no radiation and can be repeatable, which has a high‐cost performance index.

Studies have found that Point‐of‐Care Ultrasound (POCUS) as an emergency medical clinical assessment is an important method for critically ill and injured patients.^2^, ^3^, ^4^ Increasing evidence have proven that ultrasound technology can monitor cerebral arterial blood flow velocity in a rat model of cerebral hemorrhage^5^, ^6^ and is of great value for early intervention before irreversible ischemic neurological deficits.^7^ Moreover, a previous study has shown that transcranial Doppler (TCD) can detect subarachnoid hemorrhage (SAH)^8^ and the influence of ultrasonic contrast agents in cerebral vascular diagnosis.^9^ More and more evidence showed that brain ultrasound has become a reliable technology for studying neonatal brains,^10^ which can mainly detect intraventricular hemorrhage (IVH), periventricular hemorrhagic infarction, posthaemorrhagic ventricular dilatation, cystic periventricular leukomalacia (cPVL), and so forth. It is of inestimable value in the evaluation of lung, pleural, and mediastinal diseases in children.^11^

**Figure 1:**
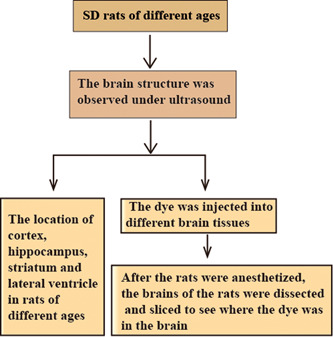
Study flowchart. To determine the location of the cortex, hippocampus, lateral ventricle, and striatum of rats of different ages, the location of the brain structure of rats was detected by ultrasound. The puncture was performed under the ultrasonic locator. After the dye was injected into the cortex, hippocampus, lateral ventricle, and striatum, the brain tissue was taken after anesthetizing the rats, frozen sections were made, and the puncture location was compared. [Color figure can be viewed at wileyonlinelibrary.com]

Puncture is widely used in animal experiments, which involves puncturing into the body cavity, organs, and medullary cavity of animals to remove the contents or inject drugs to achieve therapeutic effects. In neurological diseases, the blood−brain barrier has become the main barrier for drug delivery to the brain, and drug injection has become the focus of treatment. However, despite a large number of studies that have performed puncture directly according to the position of the brain structure and the stereotactic coordinates of the brain, which cannot effectively deliver therapeutic substances into the brain due to the lack of complete intracerebral location, it is hard and restricted to apply technical neuroscience techniques to infant rat's brain. Therefore, this experiment used the advantages of ultrasound to probe the brain structure of experimental animals. In neurological diseases, drugs delivered to several brain regions, such as the olfactory bulb, lateral ventricles, cerebral cortex, striatum, and hippocampus, can affect cognitive function and behavior in different neurodegenerative diseases.^12^, ^13^ Under the guidance of ultrasound, the location of a puncture could be determined, and the structure of the cerebral cortex, hippocampus, lateral ventricle, and striatum of rats could be observed, which provides a theoretical basis for the application of ultrasound in animal research. The flow chart of this study is displayed in Figure 1.

## 2 MATERIALS AND METHODS

### 2.1 Animals and grouping

A total of 25 normal rats were used in this experiment, including 10 rats, which were used to observe the definition of brain structure at 2‐15 d, and 5 rats at 8, 10, and 13 d to determine the location of the striatum of the corticohippocampal lateral ventricle by ultrasound and to carry out puncture injection of dye. The experimental animals were provided by the specific pathogen‐free room of the Experimental Animal Department of Kunming Medical University. The study and all procedures were approved by the Animal Ethics Committee of Kunming Medical University on March 2, 2022 (approval no. kmmu20220748). Animal care and all experimental procedures were in compliance with the “Guidelines for the Care and Use of Laboratory Animals” issued by the National Institutes of Health. Animals were bred in an environment of 18−26°C temperature, 40%−70% humidity, and a 12‐h light−dark cycle. All rats ate and drank freely.

### 2.2 Extracranial ultrasound in normal rats

All rats were anesthetized by inhalation of 5% isoflurane. The rats were placed in the prone position. The head of the rat was coated with a medical ultrasonic couplant, the sensor was perpendicular to the head, and the image was obtained from the coronal position. The parameter setting of two‐dimensional ultrasounds showed mechanical index (MI) ≤ 0.88, thermal index of soft tissues (TIS) ≥ 0.51, probe model L10LC, 27HZ, B gain 66 dB, frequency H7.0 Mhz, sound power 90%, focus number 1, focal length 2, frame correlation 4, smoothing 0, edge enhancement 0, B grayscale 8, dynamic range 272, width 20 mm, high linear density, B false color 0, speckle suppression 4, deflection 0, rotation 0°, and high‐density composite low. During the entire operation, the gain and depth were optimized and continuously adjusted. The machine type was Apogee5300, and the instruments were provided by Shantou Institute of Ultrasonic Instruments Co., Ltd. (SIUI).

### 2.3 Rats were punctured under stereotaxic apparatus

The rat cortex, hippocampus lateral ventricle, and striatum were accurately obtained according to the above‐mentioned ultrasound procedure. After anesthetized by inhalation of 5% isoflurane, rats were weighed and then placed in a small‐animal stereotaxic instrument (RWD Life Science Co. Ltd., Model: 518094). Then, the rats' heads were secured using medical tape and placed in a prone position. Disinfection of the brain and skin of rats was performed. The position of the fontanelle was accurately obtained, and direct puncture was performed according to the coordinates of the rat cortex, hippocampus, lateral ventricle, and striatum obtained by ultrasound. A microinjector (Hamilton, Model: 7635‐01/00) was used to slowly inject 3 μl) of the dye into the marked positions. The speed was 1 μL/minutes (min), and the duration was 3 min in total. After the injection, the needle was left for 2 min and taken out. Finally, the skin where the needle was inserted was disinfected with an iodophor.

### 2.4 Brain tissue sampling

The rats were anesthetized with 5% isoflurane after injection and then their limbs were fixed. After that, a scissor was used to cut the skin and muscle tissues from the back of the occipital bone, and the muscles and meninges were removed. The skulls were cut open with rongeurs from the foramen magnum to expose the brains, and the brains were taken out with tweezers and placed in a Petri dish. After the brains were prefrozen at −20°C, they were placed again on the cryostat for whole‐brain sectioning. The slice thickness was 1.5–2 mm. Finally, the pictures were observed and collected.

### 2.5 Statistical analysis

All data are presented as the primary data or mean ± standard deviation. Statistical analysis was performed using SPSS 20.0 software. One‐way analysis of variance with Tukey's post hoc test was applied for comparisons among multiple groups. GraphPad Prism software version 8.0 (GraphPad Software Inc.) was used for quantification histogram generation. Any difference with a *p* < 0.05 was considered significant.

## 3 RESULTS

### 3.1 Dynamic ultrasound detection of brain structure clarity in rats

**Figure 2:**
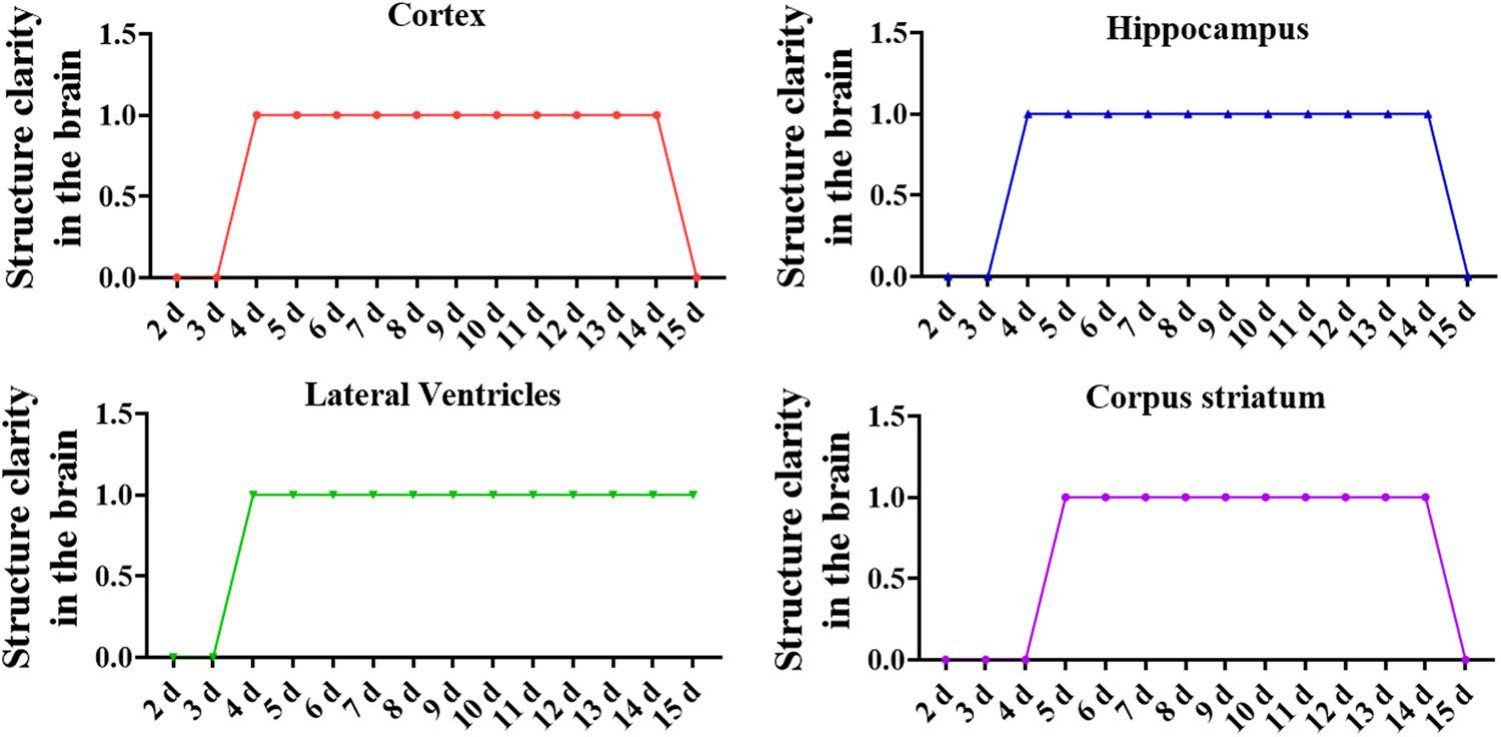
Coronal dynamic exploration of rat brain structure clarity. Coronal ultrasound was used to detect the structural clarity of the cerebral cortex, hippocampus, lateral ventricle, and corpus striatum; 1 represents clear brain structure and 0 means that the brain structure is not clear. d, days. [Color figure can be viewed at wileyonlinelibrary.com]

Because the rats' weight changes quickly in the early growth stage, body and brain sizes can vary significantly depending on the rats' weight. During the first 4 d of life, the brain tissue cannot be identified by ultrasound because the brain is small and incompletely developed. As the brain size of rats gradually increases, the skull and cerebral cortex of rats gradually become thicker after 14 d of life, and the brain structure can not be detected by ultrasound. Our study used ultrasound to detect the location of brain structures in rats of different ages to determine the location of the cortex, hippocampus, lateral ventricle, and striatum (Figure 2). From the coronal ultrasound images, it can be seen that the structures of the rat cortex and hippocampus were not clear at 2, 3, and 15 d. The definition of the lateral ventricle was higher than that of other brain structures, and the striatum was clearly structured from Days 5 to 15. Subsequently, under ultrasound combined with stereotaxic apparatus, the cortex, hippocampus, lateral ventricle, and striatum were punctured, and the dye was injected into them. After the rats were anesthetized, the brain tissues were taken, frozen‐sectioned, and the puncture positions were easily recognized.

### 3.2 Brain structure of the cortex, hippocampus, striatum, and lateral ventricle in different ages of rats under ultrasound

**Figure 3:**
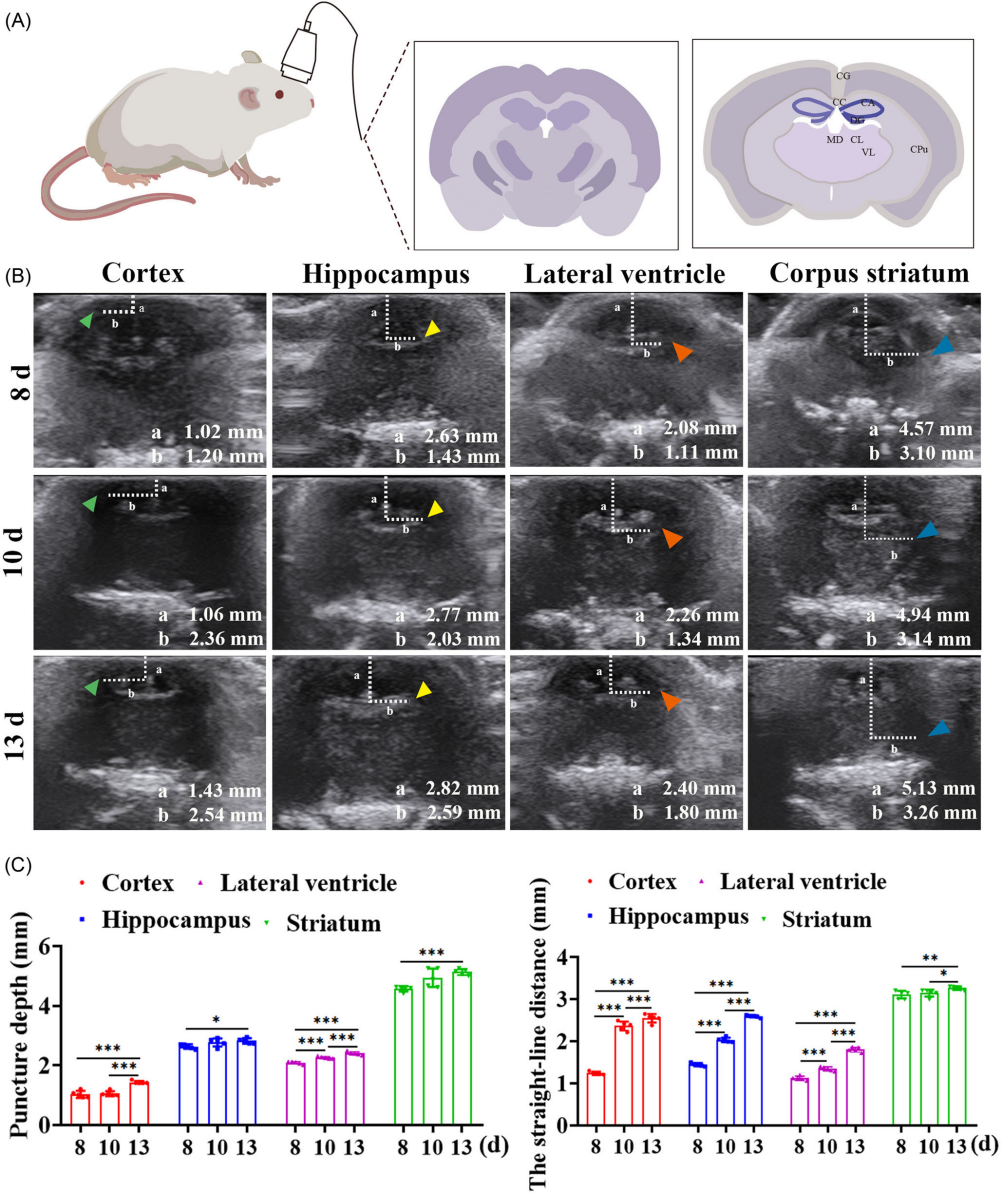
Ultrasound‐guided localization of puncture in different brain regions. (A) Schematic diagram of the brain structure of rats under ultrasound. (B) Cortex, hippocampus, lateral ventricle, and striatum images of normal rats under ultrasound. a Puncture depth. b Straight‐line distance from the anterior midline to the puncture point. 8, 10, and 13 d represent the age of normal rats. The green arrow points to the cortex; the yellow arrow points to the hippocampus; the red arrow points to the lateral ventricle; and the blue arrow points to the striatum. (C) Quantified data including the puncture depth and the straight‐line distance from the anterior midline to the puncture point. CA: hippocampus; CC: cerebral cortex; CG: cingulum; CL: centrolateral thalamic nucleus; CM: central medial thalamic nucleus; Cpu: caudate putamen; d: days; DG: dentate gyrus; MD: mediodorsal thalamic nucleus; VL: ventrolateral thalamic nucleus; VPM: ventral posterolateral thalamic nucleus. *p < 0.05; **p < 0.01; ***p < 0.001. [Color figure can be viewed at wileyonlinelibrary.com]

Ultrasound was used to determine the brain structure of rats at 8, 10, and 13 d (Figure 3A). Combined with the rat brain atlas, we can clearly see the location of the cortex, hippocampus, striatum, and lateral ventricle of rats on 8, 10, and 13 d. There were five rats in each group of experiments, and the mean ± standard deviations for accurate measurements of the puncture depth and distance from the fontanelle to the puncture point are in Table 1. It was displayed that the cortical puncture depth was the smallest and the striatum puncture distance was the largest (Figure 3B), green arrows point to the cortex, yellow arrows point to the hippocampus, red arrows point to the lateral ventricle, and blue arrows point to the striatum. With the increasing age and weight of rats, the distance between the lateral ventricle and the hippocampus from the anterior midline increased, and the puncture depth of the cortex and striatum also increased (Figure 3C, *p* <0.05). Under the guidance of ultrasound, the changes in the brain tissue of rats at different ages can be dynamically observed, providing accurate location and maximum help for subsequent puncture injection, reducing experimental errors, improving experimental efficiency, and realizing the maximum benefit of ultrasound.

**Table 1:** Location of brain structures in rats under ultrasound guidance (*n* = 5).

	Cortex			Hippocampus			Lateral ventricle			Corpus striatum		
	8 d	10 d	13 d	8 d	10 d	13 d	8 d	10 d	13 d	8 d	10 d	13 d
The puncture depth (mm)	1.02 ± 0.12	1.02 ± 0.08	1.43 ± 0.05	2.63 ± 0.07	2.77 ± 0.14	2.82 ± 0.09	2.08 ± 0.04	2.26 ± 0.03	2.40 ± 0.06	4.57 ± 0.09	4.94 ± 0.31	5.13 ± 0.10
Anterior distance (mm)	1.20 ± 0.04	2.36 ± 0.11	2.54 ± 0.10	1.43 ± 0.04	2.03 ± 0.06	2.59 ± 0.03	1.11 ± 0.05	1.34 ± 0.05	1.80 ± 0.06	3.10 ± 0.09	3.14 ± 0.09	3.26 ± 0.04
Weight (g)	17.12 ± 0.01	22.3 ± 0.01	24.61 ± 0.02	17.2 ± 0.02	22.3 ± 0.01	24.32 ± 0.01	17.2 ± 0.03	22.2 ± 0.01	24.6 ± 0.03	17.2 ± 0.02	22.12 ± 0.01	24.13 ± 0.02

*Note*: Ultrasound was used to locate the locations of the cortex, hippocampus, lateral ventricle, and striatum in rats, including puncture depth and the straight‐line distance from the anterior midline to the puncture point.

### 3.3 Rats were punctured with ultrasound‐guided stereotactic apparatus for 8 days

**Figure 4:**
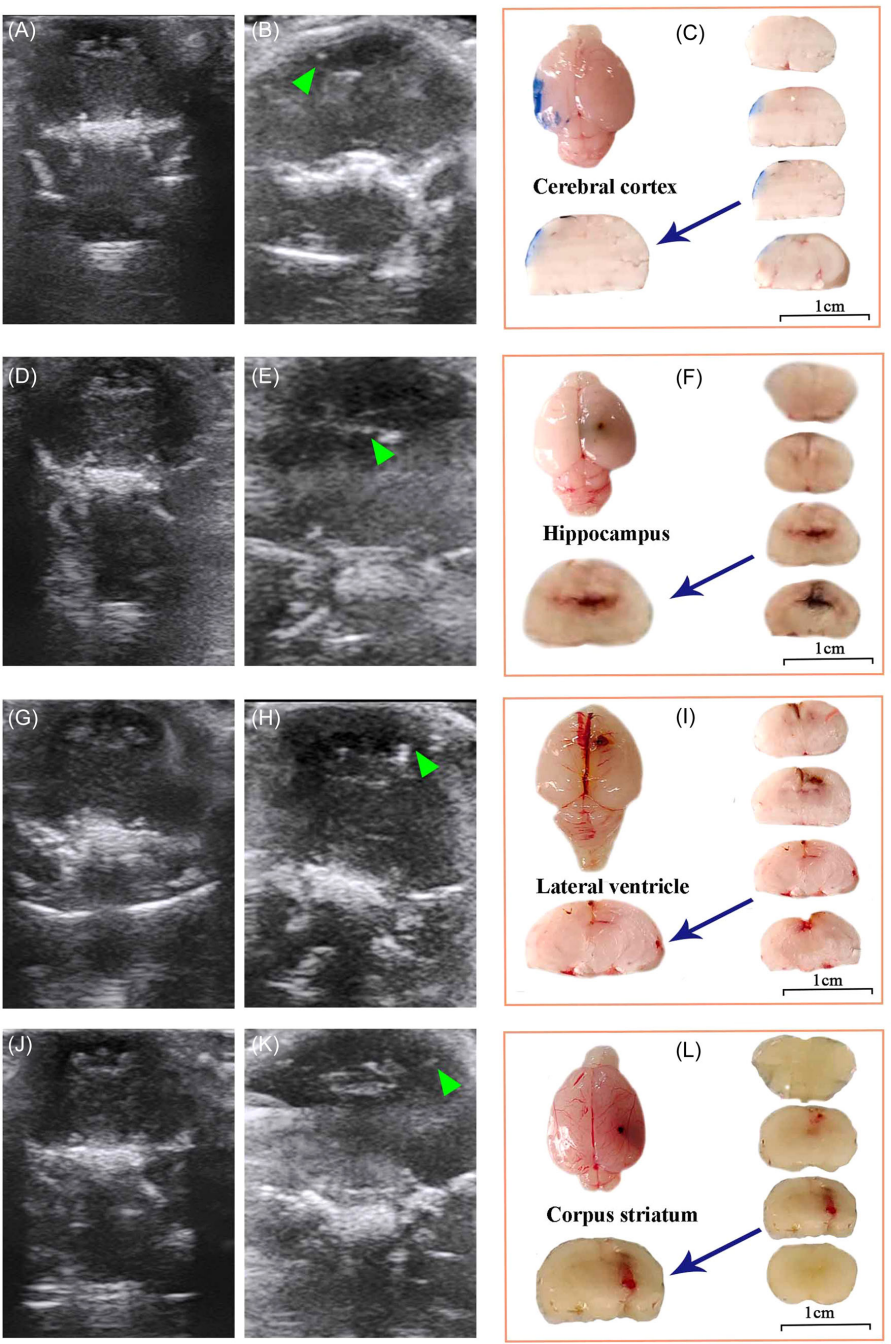
Localization images of the cortex, hippocampus, lateral ventricle, and striatum in the rat brain structure under ultrasound. (A, D, G, L) Coronal images of rats under ultrasound; images of the cortex (B), hippocampus (E), lateral ventricles (H), and striatum (M); coronal images of the brain section, dye‐stained position: cortex (C), hippocampus (F), lateral ventricle (I), and striatum (N). The scale bar of the brain section is 1 cm. [Color figure can be viewed at wileyonlinelibrary.com]

Ultrasound was used to obtain the coronal image of the rat brain, including the cortex, hippocampus, lateral ventricle, and striatum (Figure 4A,D,G,L). The distances from the anterior midline and the depth of the needle were obtained, and the location of the rat brain bregma was marked. The rat was punctured with a stereotaxic apparatus, and the puncture position was observed by ultrasound. There was a bright spot under ultrasound, which showed the position of the green arrow in Figure 4B,E,M,H. The results showed that under ultrasound, the coronal image can clearly identify the normal brain structure. The puncture depths of the cortex, hippocampus, lateral ventricle, and striatum are 1.02, 2.63, 2.08, and 5.13 mm, respectively. In addition, the whole brain and slices were displayed, and the third slice of the coronal position was magnified. It was found that the dye was accurately located on the cortex, hippocampus, lateral ventricle, and striatum. With the aid of ultrasound, the puncture can be more accurate and convenient (Figure 4C,F,I,N).

## 4 DISCUSSION

**Figure 5:**
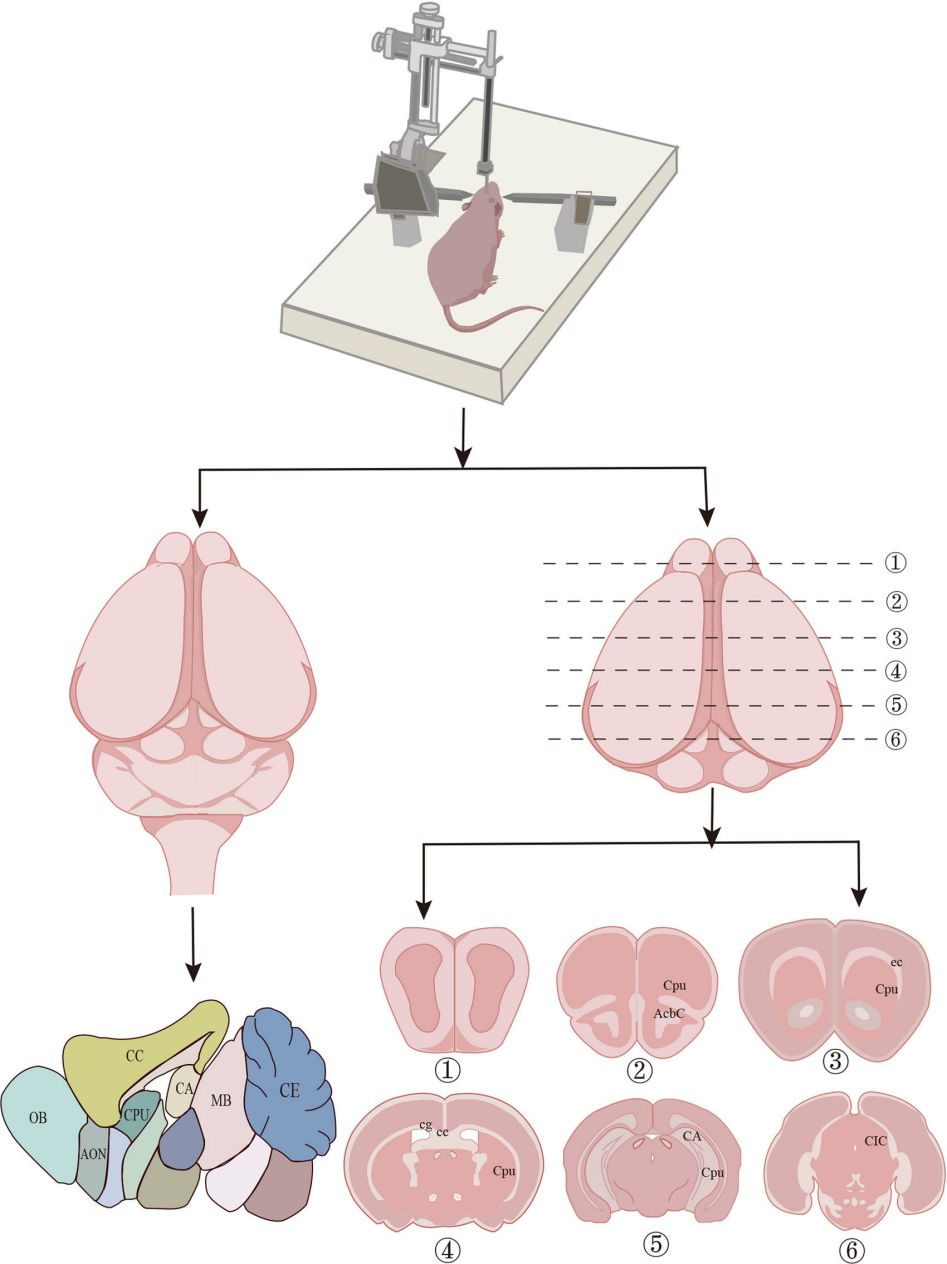
Example diagram of the research. Anatomy under ultrasound‐guided puncture. The research route of the cortex, hippocampus, lateral ventricle, and striatum puncture positions of rats of different ages under ultrasound. ①–⑥ represent coronal sections of brain anatomy in SD rats. AcbC: accumbens nucleus, core; AON: ameror olfactory nucleus; CA: hippocampus; CC: cerebral cortex; CE: central amygdaloid nucleus; cg: cingulum; CIC: Central nucleus of inferior colliculus; Cpu: caudate putamen; MB: mamillary body; OB: Olfactory Bulb. [Color figure can be viewed at wileyonlinelibrary.com]

With the aid of ultrasound, the position of the cortex, hippocampus, lateral ventricle, and striatum of normal rats can be accurately measured, and the puncture under the guidance of ultrasound images can be used to improve the accuracy of the puncture position after the rats are taken (Figure 5). Moreover, as the age of rats changes, the positions of the cortex, hippocampus, lateral ventricle, and striatum also change.

The complexity of neurological diseases and population aging poses great challenges; to get to know the changes that occur in the brain structures, it is of great importance to have excellent knowledge of the cerebral anatomy. Traditionally, histology and microscopy techniques are used to study neurological diseases, but they do not provide an imaging system for dynamic observations. So, some conventional techniques are used to study peripheral neuropathy such as magnetic resonance imaging and ultrasound. Although magnetic resonance imaging was accurate and had good resolution, considering the gold standard imaging technique to follow‐up on brain lesions, it has some questions, including contrast‐related side effects, limited accessibility, and high costs.^14^ For this reason, the brain anatomy information provided by ultrasound is helpful for the study of neurological diseases. Ultrasound imaging proved to be a viable tool for the noninvasive study of rat brain anatomy.^15^ Taking advantage of the high safety and no radiation of ultrasound, this experiment studied the brain structure of newborn rats. Because the cranium of newborn rats is relatively thin, important brain structures such as the cortex, lateral ventricle, hippocampus, and striatum under ultrasound can be clearly identified, and the position and puncture could be guided to determine the brain structure. It has been found that at 6 months of age, the horizontal distance from the anterior midline to the hippocampus is about 3.6 mm,^16^, ^17^ and the horizontal distance from the anterior midline to the lateral ventricle is 1.2 mm in 7‐day rats.^18^ Since the body weight of the pup will change rapidly during early growth, the size of the body and brain will vary greatly depending on the weight of the pup. In our study, it was possible to accurately locate brain structures with different weights and ages, which makes the experimental error smaller and the experimental data more accurate. However, the dye was injected under the stereoscope and the injection site was observed by ultrasound. It was found that the dye would spread in the required injection site. In neurological diseases, the direct intracranial injection has been used in animal experiments to treat neurological diseases, which cannot effectively deliver sufficient drugs to the brain tissue. The use of ultrasound can directly observe the diffusion of drugs into the brain tissue, ensuring the safety of experimental animals. Thus, ultrasonics‐assisted experimental research plays a great role.

Ultrasound is a noninvasive, low‐cost, high‐efficiency, and safe technology. Its versatility makes it an important tool for assisting brain structure puncture. Ultrasound also plays an important role in the diagnosis and treatment of some diseases. Studies have shown that ultrasound can be used to diagnose acute respiratory failure, acute circulatory failure disease (Lichtenstein) and identify various structural abnormalities (tendinitis, bursitis, and synovitis). It also can help to record tenderness during specific tendon processes.^19^ Bedside pulmonary ultrasound has high diagnostic value in the diagnosis of chronic obstructive pulmonary disease and cardiogenic pulmonary edema.^20^, ^21^ Moreover, studies have found that ultrasound images can guide cancer treatment,^22^ such as topical, sonodynamic therapy (SDT), and focused ultrasound surgery.^23^ In recent studies, ultrasound is considered to be a noninvasive method for screening intracranial hypertension in children with acute and severe traumatic brain injury,^24^, ^25^ and it is an effective tool for evaluating intracranial pressure and vasospasm.^26^, ^27^ It also plays an important role in the diagnosis and treatment of vasospasm after aneurysmal subarachnoid hemorrhage.^28^, ^29^ Furthermore, there are studies using ultrasound to repattern the thrombotic femoral artery in dogs, gaining good results.^30^

However, despite the advantages of ultrasound, it currently has certain limitations. This technique requires the knowledge and expertise of a well‐trained operator.^2^ Therefore, particular caution is necessary when acquiring and interpreting images. The ultrasound parameters also need to be standardized, because some studies fail to report the intensity, duration, and mechanical index used, which hinders the reproducibility of the study.^31^ Another limitation of ultrasound is that it reduces penetration in the presence of bone structure.^32^ The evaluation of pleural effusion and the performance of ultrasound‐guided pleural puncture require basic ultrasound technical expertise.^33^ In some studies, this limitation is offset by thinning or removing the skull,^34^ which causes additional damage. As a result, in this study, ultrasound imaging parameters were all standardized and were performed by professional operators to obtain accurate and repeatable images.

## 5 CONCLUSION

Due to the different ages of rats and the differences between individuals, the position of the brain structure has changed. Ultrasound can be used to monitor the brain structure of neonatal rats in real time and noninvasively. The results of this study indicate that ultrasound can dynamically and accurately detect the brain structure of 5–15‐d rats, and the ultrasound image could guide the stereotactic puncture, which can reduce experimental errors and improve the accuracy of the experimental results.

## AUTHOR CONTRIBUTIONS

Rui‐Fang Ma, Ping‐Chieh Pao and Lin Zhang interpreted the data and arranged the figures. Kun Zhang and Jin‐Xiang Liu were involved in the overall design and supervision of the work. All authors have read and approved the final version of the manuscript. [Correction added on 7 December 2023 after first online publication: This section was revised at the request of authors.]

## CONFLICT OF INTEREST STATEMENT

Kun Zhang is employed by the company Shantou Ultrasonic Instrument Research Institute Co. Ltd. He confirms that he has no commercial interest that could be produced from this manuscript. The remaining authors declare that the research was conducted in the absence of any commercial or financial relationships that could be construed as a potential conflict of interest.

## ETHICS STATEMENT

The study and all procedures were approved by the Animal Ethics Committee of Kunming Medical University (approval no. kmmu20220748).

## Data Availability

The data used and/or analyzed during the current study are available from the corresponding author up on reasonable request.
